# Reproducible Cancer Biomarker Discovery in SELDI-TOF MS Using Different Pre-Processing Algorithms

**DOI:** 10.1371/journal.pone.0026294

**Published:** 2011-10-14

**Authors:** Jinfeng Zou, Guini Hong, Xinwu Guo, Lin Zhang, Chen Yao, Jing Wang, Zheng Guo

**Affiliations:** 1 Bioinformatics Centre, School of Life Science, University of Electronic Science and Technology of China, Chengdu, People’s Republic of China; 2 College of Bioinformatics Science and Technology, Harbin Medical University, Harbin, People’s Republic of China; Queen Elizabeth Hospital, Hong Kong

## Abstract

**Background:**

There has been much interest in differentiating diseased and normal samples using biomarkers derived from mass spectrometry (MS) studies. However, biomarker identification for specific diseases has been hindered by irreproducibility. Specifically, a peak profile extracted from a dataset for biomarker identification depends on a data pre-processing algorithm. Until now, no widely accepted agreement has been reached.

**Results:**

In this paper, we investigated the consistency of biomarker identification using differentially expressed (DE) peaks from peak profiles produced by three widely used average spectrum-dependent pre-processing algorithms based on SELDI-TOF MS data for prostate and breast cancers. Our results revealed two important factors that affect the consistency of DE peak identification using different algorithms. One factor is that some DE peaks selected from one peak profile were not detected as peaks in other profiles, and the second factor is that the statistical power of identifying DE peaks in large peak profiles with many peaks may be low due to the large scale of the tests and small number of samples. Furthermore, we demonstrated that the DE peak detection power in large profiles could be improved by the stratified false discovery rate (FDR) control approach and that the reproducibility of DE peak detection could thereby be increased.

**Conclusions:**

Comparing and evaluating pre-processing algorithms in terms of reproducibility can elucidate the relationship among different algorithms and also help in selecting a pre-processing algorithm. The DE peaks selected from small peak profiles with few peaks for a dataset tend to be reproducibly detected in large peak profiles, which suggests that a suitable pre-processing algorithm should be able to produce peaks sufficient for identifying useful and reproducible biomarkers.

## Introduction

Proteomic technologies based on mass spectrometry (MS) [1] have increasingly become the method of choice for the identification of biomarkers that are useful for differentiating diseased and normal samples [2], [3], [4]. However, similar to microarray studies [5], [6], the use of MS techniques to identify disease biomarkers has been hindered by irreproducibility [7], [8]. For example, the biomarkers identified in four prostate cancer studies are very different [8]. Recently, Callesen *et al*. [7] showed that only 10 of 207 biomarkers reported in 15 MS-based breast cancer studies were detected in more than 2 studies. This irreproducibility raises questions about the biological significance and clinical implications of the detected biomarkers.

Many factors, such as sample processing and operating procedures for the experiments, can affect the reproducibility of disease biomarkers [9], [10], [11], [12], [13], [14], [15]. Importantly, the data pre-processing algorithm chosen to produce peak profiles may greatly affect biomarker identification [16]. Some studies have attempted to find the optimum pre-processing algorithm for detecting peaks [17], [18], [19]. However, until now, no widely accepted agreement has been reached. For example, based on simulated data with predefined true peaks, Cruz-Marcelo *et al*. [17] and Emanuele *et al*. [18] evaluated several algorithms in terms of both sensitivity (defined as the proportion of true peaks that were correctly identified) and specificity (defined as the false discovery rate (FDR)). These two studies reached different conclusions on the three algorithms that they both evaluated, which were MassSpecWavelet [20], Cromwell [21] and commercial software produced by Ciphergen Biosystems. Cruz-Marcelo *et al*. [17] reported that these algorithms offered high sensitivity with a low FDR, whereas Emanuele *et al*. [18] showed that they had low sensitivity and a low FDR. This conflict could have been introduced by differences in their simulation data, which in general tend to be biased to specific scenarios. A solution for avoiding bias is to adopt real data instead of simulated data. Unfortunately, with real data, the sensitivity and FDR of an algorithm cannot be evaluated because the true peaks are unknown. However, pre-processing algorithms can be compared in terms of peak detection reproducibility by assessing peak overlap. Notably, reproducibility is a critical measure for validating biological discoveries that is distinct from sensitivity and specificity [6], [22], [23], [24].

In this study, using real prostate and breast cancer data, we first evaluated the reproducibility of peak detection among three widely used pre-processing algorithms that detect peaks dependent on the average spectrum of all of the spectra (see *Methods*), including SpecAlign [25], MassSpecWavelet [20] and Cromwell [21]. More importantly, we further evaluated the reproducibility of detection of differentially expressed (DE) peaks (often defined as biomarkers), which has been a focus of the biological community but have not been fully evaluated with either simulated or real data. Our results indicate that the number of peaks detected for a dataset varies dramatically depending on the pre-processing algorithm. Our results also revealed two important factors affecting the consistency of DE peak identification using different pre-processing algorithms. The first factor is that a peak profile may lack DE peaks found in another profile, which can affect reproducibility before the selection of DE peaks. The second factor is that a large peak profile with many peaks may suffer from low statistical power for identifying DE peaks because of the large scale of the test together with small sample number [26], [27], [28], [29]. Fortunately, our results indicate that the power of large peak profiles can be increased by the stratified FDR control approach [30]. Consequently, DE peaks selected from small peak profiles tend to be reproducibly detected in large peak profiles. Based on the analysis of this study, we suggest that a suitable pre-processing algorithm should be able to produce peaks sufficient for the identification of useful and reproducible biomarkers.

## Materials and Methods

### Cancer datasets

The prostate cancer data, which was downloaded from http://www.evms.edu/vpc/seldi/, consisted of duplicate spectra for 168 cancer and 81 normal serum samples measured by SELDI-TOF MS (IMAC-3 chips), with the mass-to-charge (*m/z*) ratio ranging from 0 to 200 kDa [31]. The blood samples of diagnosed stage I-IV patients were procured from the Department of Urology, Eastern Virginia Medical School and the samples of healthy men were obtained from free screening clinics open to the general public (see details in [31]). The serum samples were obtained from the Virginia Prostate Center Tissue and Body Fluid Bank. The breast cancer data, which was downloaded from http://bioinformatics.mdanderson.org/pubdata.html, consisted of duplicate spectra for 26 cancer and 14 normal plasma samples measured by SELDI-TOF MS (IMAC-Cu chip), with the *m/z* ratio ranging from 10 to 100 kDa [32]. The blood samples were obtained from diagnosed stage I-III breast carcinoma patients and healthy volunteers (see details in [32]). The plasma samples were conducted at the Nellie B. Connally Breast Center at the University of Texas M. D. Anderson Cancer Center.

For each pair of duplicate spectra, the two spectra were pre-processed separately and then averaged to produce a consensus profile. Considering measurement noise and detection limitations, we only used those peaks in the *m/z* range of 1–10 kDa for breast cancer and 2–40 kDa for prostate cancer in our analyses as in the original papers [31], [32].

### Data pre-processing algorithms

**Figure 1:**
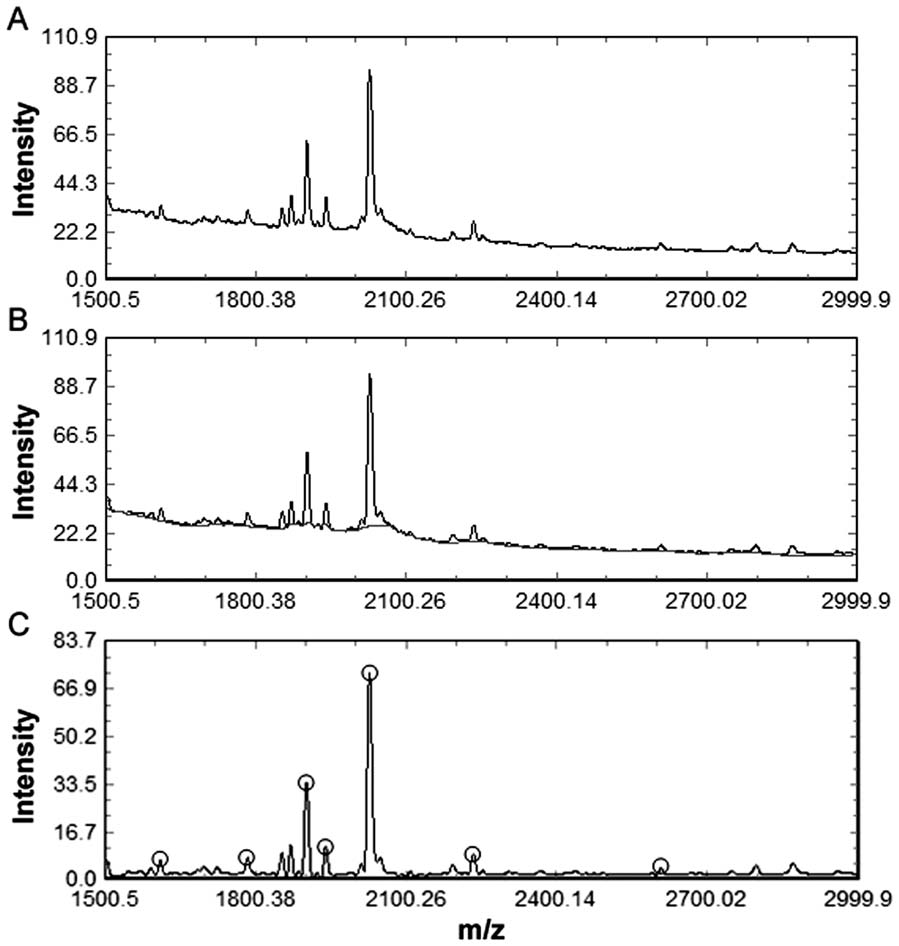
Illustration of a pre-processing procedure. (A) Raw spectrum. (B) Smoothed spectrum. The baseline estimated from the smoothed spectrum is represented as the gray line. (C) Normalized spectrum. The baseline is subtracted from the smoothed spectrum. Then, the baseline-subtracted spectrum is normalized. The peaks detected based on the normalized spectrum appear in circles.

As illustrated in Figure 1, SELDI-TOF-MS data are usually pre-processed by multiple steps including denoising (smoothing), baseline subtraction, normalisation, peak detection, clustering of peaks and peak quantification [17]. The three algorithms analysed in this study detect peaks according to the average spectrum of all the spectra, and the pre-processing procedures are described below. The specific parameter settings used for each algorithm can be found in Text S1.

(1). SpecAlign [25] pre-processes data as follows: a) spectrum smoothing using the Savitzky-Golay filter; b) subtracting the baseline estimated by a restrained moving average; c) rescaling intensities to positive values by making the minimum value 0; d) normalising intensities to let all spectra have the same total ion current; e) generating an average spectrum; f) using the fast Fourier transform (FFT)/peak matching combined method to align the detected peaks of individual spectra to those identified in the average spectrum; and g) picking peaks. The default height ratio that served as the signal-to-noise ratio (SNR) was 1.5.

(2). The MassSpecWavelet package for peak detection [20] combined with the PROcess package for peak quantification [33] (denoted MSW/PRO). MassSpecWavelet has been reported to have high sensitivity with a low FDR for peak detection [17]. However, it does not quantify the detected peaks. Thus, based on work by Cruz-Marcelo *et al*. [17], we used PROcess to quantify peaks detected by MassSpecWavelet. MassSpecWavelet detects peaks using the continuous wavelet transformation on the average spectrum of all of the spectra. For each spectrum, PROcess subtracts the baseline, which is estimated by linear interpolation, then normalises the intensities using the median area under the curves of all of the spectra, and finally quantifies the detected peaks of individual spectra by the local maximum within the predefined interval. The default SNR for peak detection was 3.

(3). Cromwell [21] pre-processes data by a) computing an average spectrum; b) denoising the average spectrum by the undecimated discrete wavelet transform; c) correcting intensities for the average spectrum by subtracting the baseline, which is estimated by a monotone minimum curve; d) finding peaks with local maximal intensities for the average spectrum; e) repeating b) and c) for each spectrum, normalising intensities with average total ion current, and quantifying peak intensities using the maximum within the intervals defining peaks on the average spectrum; and f) extracting peaks with a user-defined SNR. The default SNR was set at 5, according to the recommendation of the developers.

The output of a pre-processing algorithm is a peak profile for the dataset, which is composed of the detected peaks and their corresponding intensities in each spectrum. For simplicity, the peak profiles produced by SpecAlign, MSW/PRO and Cromwell are denoted SpecAlign profile, MSW/PRO profile and Cromwell profile, respectively.

Two peaks with a *m/z* ratio difference within a shift range may correspond to the same biological molecule [17], [34]. In this study, we used shift ranges of ±0.1%, ±0.2% and ±0.3%, and the results were similar. For simplicity, we only present the results based on the commonly used shift range of ±0.3% [17], .

Because the optimisation goals for peak detection are not defined in real data, the default parameter settings for pre-processing algorithms are used for detecting peaks in most applications. However, some studies may tune the SNR to find more or less peaks [17], [18], [35], [36]. Thus, we similarly tuned the SNR in our study to compare pre-processing algorithms. In addition, because a lower SNR may detect more true and useful peaks, we mainly considered the lower of the two SNRs when comparing one algorithm with another (see details in the *Discussion*).

### Detection of DE peaks and consistency scores

Student’s *t*-test was used to evaluate the significance of the differences between the intensity means of the cancer and normal samples. For multiple testing correction, we used the Benjamini-Hochberg procedure to control the FDR at a given level [37].

The consistency of two peak lists was measured by the PO (percentage of overlaps) score [38]. Supposing list 1 with *l*_1_ peaks and list 2 with *l*_2_ peaks share *k* peaks, then the PO score from list 1 (or 2) to list 2 (or 1) is *PO*_12_ =  *k*/*l*_1_ (or *PO*_21_ =  *k*/*l*_2_). Because the PO score depends on the list lengths, we also calculated the normalised PO score (*n*PO), which is defined as the proportion of the observed score beyond chance to the corresponding maximum potential score beyond chance [38]:
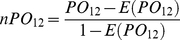
(1)

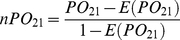
(2)where E(PO_12_) (or E(PO_21_)) was estimated as the average of the PO_12_ (or PO_21_) scores for 1,000 pairs of peak lists (with lengths *l*_1_ and *l*_2_) extracted randomly from the two raw *m/z* lists. Among the scores for the 1,000 random pairs of peak lists, the *p*-value of observing the PO score by random chance was calculated as the proportion of the scores not less than the observed score.

The PO (*n*PO) score between two lists of DE peaks was calculated by the same approach as described above, except that a DE peak was defined as being shared by two lists only if it was regulated in the same direction in both peak profiles [38]. E(PO) was evaluated using DE peak lists randomly extracted from the two peak profiles. Here, we present the PO (*n*PO) score from the shorter list to the longer list and evaluate the degree that the shorter list is covered by the longer list.

We denote the PO (*n*PO) score from the peaks detected by algorithm *A* to those detected by algorithm *B* as PO_AB_ (*n*PO_AB_), while PO^DE^_AB_ (*n*PO^DE^_AB_) is for DE peaks.

### Stratified FDR control approach

In large-scale testing with current multiple testing adjustments, the power might decrease as the number of tests increases [27], [30], [39]. To increase the power, a stratified FDR control approach has been proposed [24]. As a proof of principle, we analysed whether the consistency of DE peak detection can be increased by improving the ability to identify DE peaks in large peak profiles using the stratified FDR control approach, which is based on the assumption that peaks with large fold change (FC) values may be more likely to be true DE peaks [40]. First, we applied the k-means clustering algorithm to partition the peaks into *k* groups, by minimising the sum of the squared Euclidean distance between the FC value for each peak and its nearest cluster centre [41]. The optimal *k* was chosen as the partition resulting in a maximal mean of silhouette values, which measures how similar a peak is to other peaks in its own group compared with those in other groups [42]. Then, at a particular FDR control level, we selected DE peaks in each group. As there is no overlap between the discoveries from different groups, the FDR of the integrated results is still less than the given FDR level [30].

## Results

### Reproducibility of peak detection

**Figure 2:**
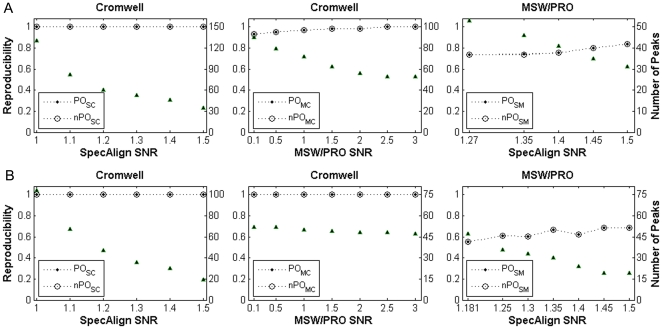
Reproducibility of peak detection across pre-processing algorithms. (A) For prostate cancer and (B) for breast cancer. Reproducibility was evaluated between one algorithm (*x*-axis label) with various SNRs and another (title) with the default SNR. The default SNRs for SpecAlign, MSW/PRO and Cromwell were 1.5, 3 and 5, respectively. The filled triangles represent the number of peaks (right *y*-axis) detected by the algorithm, which is shown by the *x*-axis label. All PO (*n*PO) scores were significantly higher than expected by chance (*p*<2.2E-11).

In the following, the results for each algorithm were based on its default SNR unless otherwise mentioned. For the prostate cancer dataset, 31 and 53 peaks were detected by SpecAlign and MSW/PRO, respectively, and all of them were included in the 420 peaks detected by Cromwell. Furthermore, we evaluated the reproducibility of peak detection using the same number of peaks by decreasing the SNR of one of the two algorithms. However, even using the lowest SNRs of 1 and 0.1 allowable for SpecAlign and MSW/PRO, respectively, only 130 and 90 peaks were detected. Most were included in the peaks detected by Cromwell with PO_SC_ (*n*PO_SC_) and PO_MC_ (*n*PO_MC_) scores as high as 1 (1) and 0.93 (0.93), respectively (Figure 2A). For the comparison between SpecAlign and MSW/PRO, the PO_SM_ (*n*PO_SM_) score was 0.84 (0.84). When the SNR was decreased to 1.27, SpecAlign detected the same number of peaks (53) as MSW/PRO, but the score decreased to 0.74 (0.73) (Figure 2A).

For the breast cancer dataset, 19 and 47 peaks were identified by SpecAlign and MSW/PRO, respectively, and all of them were included in the 287 peaks detected by Cromwell. Furthermore, as shown in Figure 2B, even after decreasing the SNR to the lowest allowable values for SpecAlign and MSW/PRO, only 104 and 52 peaks, respectively, were detected, and all of them were detected by Cromwell. The consistency score between SpecAlign and MSW/PRO was not high, with a PO_SM_ (*n*PO_SM_) score of 0.68 (0.68). After the SNR was decreased to 1.181, SpecAlign detected the same number of peaks (47) as MSW/PRO, and the PO_SM_ (*n*PO_SM_) score decreased to 0.55 (0.55) (Figure 2B).

The above results suggest that when using the default SNR for each algorithm in these two datasets, SpecAlign and MSW/PRO tend to be less sensitive at peak detection than Cromwell. All of the detected peaks also tend to be detected by Cromwell. Cromwell could still capture almost all of the peaks detected by SpecAlign and MSW/PRO when the SNRs of the latter two less sensitive algorithms were lowered.

### Reproducibility of DE peak detection

**Figure 3:**
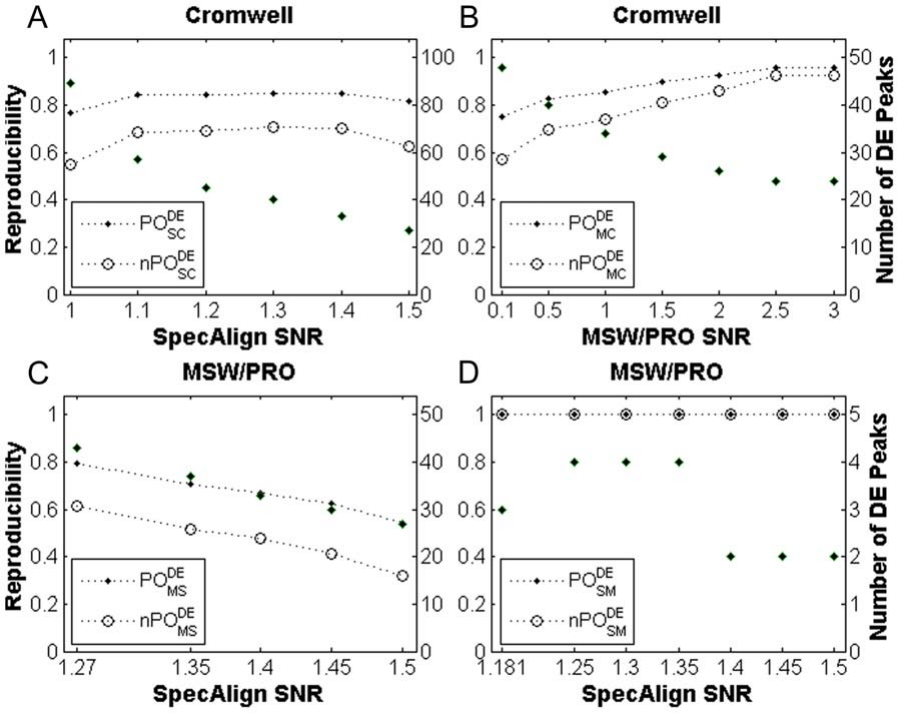
Reproducibility of DE peak detection across pre-processing algorithms. (A), (B), (C) for prostate cancer and (D) for breast cancer. Reproducibility was evaluated between one algorithm (*x*-axis label) with various SNRs and another (title) with the default SNR. The default SNRs for SpecAlign, MSW/PRO and Cromwell are 1.5, 3 and 5, respectively. The DE peaks were selected with a 10% FDR control. The filled diamonds represent the number of DE peaks (right *y*-axis) detected using the algorithm shown by the *x*-axis label. All PO^DE^ (*n*PO^DE^) scores were significantly higher than expected by chance (*p*<7.0E-3).

We then evaluated the reproducibility of DE peak identification in peak profiles produced by different pre-processing algorithms. For the prostate cancer dataset, 27 and 24 DE peaks were selected from the SpecAlign and MSW/PRO profiles, respectively, with a 10% FDR control. Most of these were also present in the 229 DE peaks identified from the Cromwell profile, and the PO^DE^_SC_ (*n*PO^DE^_SC_) and PO^DE^_MC_ (*n*PO^DE^_MC_) scores were 0.81 (0.62) and 0.96 (0.92), respectively. Although all of the peaks in the SpecAlign profile were included in the Cromwell profile, more than 10% of the selected DE peaks were not included in the DE peaks found in the Cromwell profile. After the SNRs were decreased for SpecAlign and MSW/PRO, the consistency between the DE peaks from these two peak profiles and those of the Cromwell profile decreased slightly (Figure 3A and 3B). The consistency between the 27 and 24 DE peaks detected in the SpecAlign and the MSW/PRO profiles was relatively low, with a PO^DE^_ MS_ (*n*PO^DE^_MS_) score of 0.54 (0.31). However, after the SNR was decreased for SpecAlign, the score increased to 0.79 (0.61) as more peaks were included in the enlarged SpecAlign profile and were detected as DE peaks (Figure 3C).

For the breast cancer dataset, with a 10% FDR control, only 2 DE peaks were selected from the SpecAlign profile, and they were included in the 8 DE peaks selected from the MSW/PRO profile with a PO^DE^_SM_ (*n*PO^DE^_SM_) score of 1 (1). After the SNR was decreased for SpecAlign, similar results were observed (Figure 3D). However, no DE peaks were selected from the Cromwell profile.

### Two major factors affect the consistency of DE peak identification

Our analysis revealed two major factors that can affect the consistency of DE peak identification using different pre-processing algorithms. The first factor is that some DE peaks selected from one peak profile may not be included in another peak profile. For example, for the prostate cancer dataset, with a 10% FDR control, 11 of the 24 DE peaks identified from the MSW/PRO profile were not included in the SpecAlign profile. Notably, after the SNR of SpecAlign decreased to 1.27, 6 of these 11 DE peaks were included in the SpecAlign profile and selected as DE peaks, which led to increased reproducibility (Figure 3C). Obviously this factor greatly affects the consistency of DE peak identification. The second factor is that the statistical power of identifying DE peaks in different peak profiles varies. Thus, some peaks shared by two peak profiles might be detected as DE peaks in one profile but not in another. The statistical power can be affected by many variables, such as peak quantification, the number of peaks for testing, the sample size, the proportion of true positives and the FDR control level [6], [26], [27], [28]. Here, we mainly analysed the effects of the number of tests and sample size on power.

First, we used an example to illustrate the effect of the number of tests. In the breast cancer dataset, at a 10% FDR control level, no DE peaks were detected in the whole Cromwell profile, which consisted of 287 peaks. However, when considering a subprofile of the Cromwell profile composed of all the peaks included in the MSW/PRO profile, 6 DE peaks were detected and they were all included in the 8 DE peaks identified in the MSW/PRO profile. Notably, the *t*-test *p*-value cutoff for declaring significance based on the Benjamini-Hochberg FDR procedure [37] was 0.013, but it decreased to 0.0003 in the whole Cromwell profile, which resulted in zero power for finding DE peaks (i.e., no DE peaks were found). Similarly, when considering a subprofile of the Cromwell profile composed of all of the peaks of the SpecAlign profile, 2 DE peaks were detected at the 10% FDR control level, and they were identical to the 2 DE peaks identified from the SpecAlign profile.

**Figure 4:**
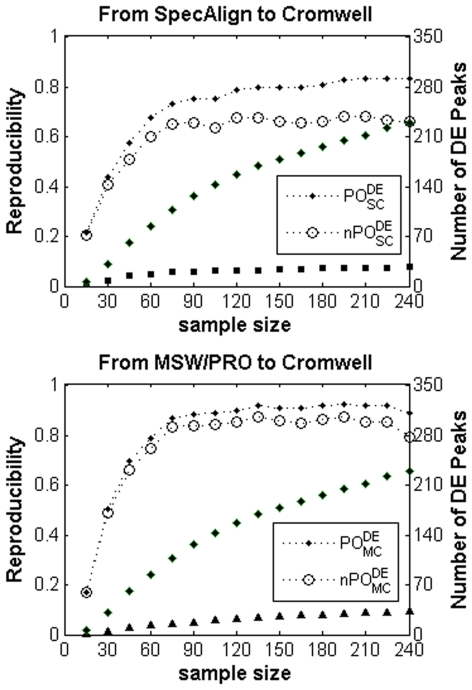
Average number of DE peaks and average PO^DE^ (nPO^DE^) score at various sample sizes for prostate cancer. The default SNR was used for each pre-processing algorithm. At each sample size, the average number of DE peaks detected at a 10% FDR control was calculated based on 100 randomly sampled subsets. The filled squares, triangles and diamonds represent the average number of DE peaks (right *y*-axis) detected using SpecAlign, MSW/PRO and Cromwell, respectively.

To illustrate the effect of sample size, we randomly sampled subsets at various sample size levels from the prostate cancer dataset of 249 samples. At each sample size level, we randomly sampled 100 subsets with the proportions of normal and cancer samples in each subset held identical to those in the raw dataset. As the sample size increased, the number of DE peaks selected with a 10% FDR control in the peak profile produced by each pre-processing algorithm increased, which indicates that the power to detect DE peaks increased (Figure 4). Consequently, the consistency of the DE peaks selected using the different pre-processing algorithms increased greatly.

### Improving reproducibility by increasing statistical power

**Figure 5:**
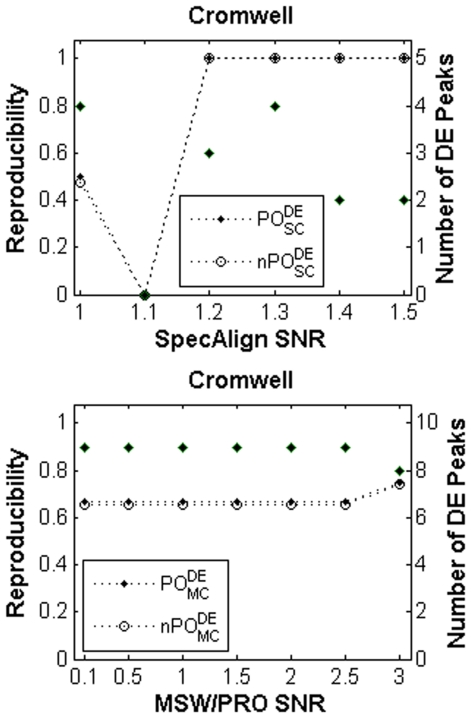
Reproducibility of DE peak detection across pre-processing algorithms. Using Cromwell at the default SNR, the stratified FDR control approach detected 16 DE peaks at the 10% level. For SpecAlign and MSW/PRO, the simple FDR control approach was used to select DE peaks. All PO^DE^ (*n*PO^DE^) scores were significantly higher than expected by chance (*p*<0.013). For a detailed description of the figures see the legend to Figure 3.

As shown above for the breast cancer dataset, the complete lack of statistical power for identifying DE peaks in some large peak profiles is an important factor affecting the consistency of DE peak detection. As a proof of principle, we demonstrated that the ability to find DE peaks in the Cromwell breast cancer profile could be improved by the stratified FDR control approach, which can increase the consistency between the identified DE peaks and those selected from the SpecAlign and MSW/PRO profiles. Using the k-means clustering algorithm as described in the *Methods*, the 287 peaks detected in the Cromwell profile were clustered into 2 groups. One group contained 259 peaks with low FC values, and the other group contained 28 peaks with high FC values. With a stratified FDR level of 10%, a total of 16 DE peaks were detected, which included most of the DE peaks detected in the SpecAlign and MSW/PRO profiles using the default SNRs with a PO^DE^_SC_ (*n*PO^DE^_MC_) and PO^DE^_MC_ (*n*PO^DE^_MC_) of 1 (1) and 0.75 (0.74), respectively. By lowering the SNRs for SpecAlign and MSW/PRO, similar results were generally obtained (Figure 5). However, after the SNR decreased to 1 for SpecAlign, the PO^DE^_SC_ (*n*PO^DE^_SC_) score was only 0.5 (0.47). This result indicates that the stratified FDR control approach can greatly increase detection power, but there is still some room for improvement.

However, with the Cromwell prostate cancer profile, the stratified and simple FDR control approaches had the same power (i.e., they detected the same DE peaks). This result may be because the power of the simple FDR control approach to identify DE peaks was already high.

### Comparison with biomarkers reported in the original breast cancer study

A total of 5 DE peaks were reported in the original study of the breast cancer dataset [32]. Briefly, the pre-processing procedure used in the original paper included the Savitzky-Golay filter, baseline subtraction, normalisation to the same total ion current and extracting peaks with SNR no less than 3.0, and the DE peaks were selected with a *t*-statistic score >3.5. We evaluated whether these 5 DE peaks could be reproduced using the three pre-processing algorithms with their default SNRs. When the SpecAlign algorithm was used, only 2 of these 5 DE peaks were detected as peaks and then detected as DE peaks at the 10% FDR control. Using the MSW/PRO algorithm, all 5 DE peaks were identified as peaks and then detected as DE peaks. Using the Cromwell algorithm, all 5 DE peaks were detected as peaks, but none was selected as a DE peak at the FDR level of 10% by the simple FDR control approach. However, all 5 DE peaks were included in the 16 DE peaks selected using the Cromwell algorithm at the FDR level of 10% when using stratified FDR control.

## Discussion

Reproducibility is of fundamental importance for the validation of biological discoveries from high-throughput data. In MS studies, pre-processing algorithms may greatly affect biomarker discovery. Using biological data for cancer, our study showed that the number of peaks identified in a dataset varies depending on the pre-processing method. It also revealed that the consistency of DE peak identification is affected by two important factors, the absence of some DE peaks in another peak profile and the reduced statistical power of DE peak identification in profiles with a large number of peaks but a small number of samples. Our findings indicate that DE peaks selected from small peak profiles tend to be reproducibly detected in large profiles when sufficient power for identifying DE peaks in large profiles is achieved through powerful statistical approaches, such as the stratified FDR control approach. The analyses in this study could be extended to other MS-based proteomic technologies. For example, for tandem mass spectrometry (MS/MS), the use of different pre-processing algorithms for peak detection and different search engines for matching proteins could produce varied protein profiles [43]. Thus, the two factors revealed in this study could also affect the consistency of biomarker detection in MS/MS studies.

Based on a simulation study, Cruz-Marcelo *et al*. [17] suggested that the combination of MassSpecWavelet and PROcess offers high sensitivity with a low FDR for peak detection. However, based on our analysis of the reproducibility of peak and DE peak detection based on two real datasets, the MSW/PRO algorithm (i.e. the combination of MassSpecWavelet and PROcess) tended to detect fewer peaks than Cromwell, which indicated that it might be less sensitive for peak detection and might miss some DE peaks detectable by using Cromwell. In addition, we evaluated the reproducibility of the three average spectrum-dependent algorithms used in this study with the widely used commercial software ProteinChip Software 3.2.1 and Biomarker Wizard (denoted as Ciphergen) for the breast cancer dataset. The required raw data from the original study were not available for Ciphergen to evaluate the prostate cancer dataset. The results showed that the Ciphergen algorithm tended to be more sensitive for peak detection than SpecAlign or MSW/PRO, and most of the peaks detected by these three algorithms also tended to be detected by Cromwell (see details in Text S1 and Figure S1); similar results were for DE peak detection (Text S1 and Figure S2). Notably, these results based on limited real data and those based on simulated data may only weakly reflect the performance of these pre-processing algorithms on data with different characteristics. Thus, we still cannot conclusively state that a specific algorithm is optimal for pre-processing all data. Nevertheless, based on our results, we can suggest a guideline for selecting a suitable pre-processing algorithm. To find useful and reproducible biomarkers, the algorithm should be able to produce sufficient peaks and achieve high sensitivity in peak detection. One problem is that a large peak profile is likely to include more random signals (false peaks), which may decrease the power of the subsequent detection of DE peaks in this profile. However, this problem can be alleviated by the use of statistically powerful approaches such as the stratified FDR control approach. In addition, increasing sample size can improve the power and, consequently, the reproducibility of DE peak detection. Thus, when sufficient power can be achieved through a powerful statistical approach or a large sample size, Cromwell can capture more biomarkers than the other pre-processing algorithms analysed in this study.

The fact that some DE peaks selected from one peak profile are not identified as peaks in another profile may suggest that these DE peaks have relatively low intensities. Thus, they might be less interesting in clinical applications. However, many biologically interesting molecules relevant to diseases are low-abundance proteins in human biofluids such as serum and plasma [44]. Some low-abundance proteins, such as the prostate-specific antigen (PSA) for prostate cancer [44] and human epidermal growth factor receptor 2 (HER2) for breast cancer [45], have been selected as clinical biomarkers. Discovering such low-abundance biomarkers is an important application of MS-based proteomic technologies [46], [47].

In addition to the factors revealed in this study, other factors may also affect the reproducibility of DE peak detection. For example, molecular isotopes with different charges could induce an improper alignment of spectra and produce multiple peaks in a spectrum [48], which could reduce the power and eventually the reproducibility of DE peak detection. Dijkstra *et al*. [48] proposed an algorithm to reduce the number of multiple-charge peaks for the underlying molecules, and this may increase the power and reproducibility of DE peak detection.

Other approaches might also improve the power of selecting DE peaks with FDR control for multiple testing. For example, by only considering peaks with large changes between diseased and normal samples, the power could increase as the number of tests decreases [40]. However, this approach considers only a portion of the total tests, and some true positives may be lost. In contrast, the stratified FDR control approach considers all of the tests. However, its performance depends on the criteria for data stratification. In addition to the simple k-means clustering algorithm used in this study, other stratification approaches, such as hierarchical clustering, could be used. Currently, finding the optimal stratification remains an open question [30], [49], [50], [51] that warrants further study.

In this study, we analysed the consistency of biomarkers identified in different peak profiles for a single MS dataset pre-processed by different algorithms. Usually, the sample handling protocol is identical for all samples in a study (see the detailed sample handling protocols for the two datasets used in this study in [31] and [32]). In this situation, the computational normalization can be applied to reduce the unknown variability of samples [52], [53]. Notably, a more challenging task is to analyse the reproducibility of biomarker discovery across different studies (datasets) for a disease [9], [11]. It is known that intensities of proteins depend on sample handling protocols. For example, the clotting time can affect the intensities of proteins related to the clotting of blood [15]. The computational normalization can not correct such variability. Therefore, the establishment of standard operating procedures for serum and plasma collection is very important for enhancing the reproducibility of SELDI data and thereby for improving the reproducibility of biomarker discovery across different studies [54]. Alternatively, an experimental normalization approach using known protein (peptides) can be applied to correct the variability induced by sample handling [55], [56]. Notably, the known proteins (peptides) need to be carefully selected to balance the trade-off between reducing the variability of the types of proteins to which they belong and increasing the intensity bias of the other types of proteins [57]. In addition, the consistency between biomarker lists identified from different studies is usually measured by counting the overlaps, such as in this study. However, observing low overlap across biomarker lists identified from different high-throughput datasets is highly likely because the sample sizes of current studies are often insufficient to fully capture large biological variations [6], [26]. Because complex diseases are often characterised by many functionally correlated molecular changes [58], [59], we have proposed consistency scores for evaluating the reproducibility of disease biomarker discovery at the systems biology level [38], [60]. In the future, by applying these consistency scores, we plan to evaluate the reproducibility of DE peaks detected in different MS-based studies for a disease, an approach that is currently limited by the fact that few MS datasets for cancer are publicly available [61].

## Supporting Information

Figure S1 **Reproducibility of peak detection between the average spectrum-dependent algorithms and Ciphergen for the breast cancer dataset.** The reproducibility was evaluated between one algorithm (*x*-axis label) with various SNRs and another (title) with the default SNR. The default SNRs for SpecAlign, MSW/PRO, Cromwell and Ciphergen were 1.5, 3, 5 and 5, respectively. The filled triangles represent the number of peaks (right *y*-axis) detected by the algorithm shown by the *x*-axis label. All PO (*n*PO) scores were significantly higher than expected by chance (*p*<7.5E-12).
(TIF)

Figure S2 **Reproducibility of DE peak detection between the average spectrum-dependent algorithms and Ciphergen for the breast cancer dataset.** For Ciphergen with the default SNR, the stratified FDR control approach detected 7 DE peaks at the level of 10%. For SpecAlign and MSW/PRO, the simple FDR control approach was used to select DE peaks. All PO^DE^ (*nc*PO^DE^) scores were significantly higher than expected by chance (*p*<9.0E-3). For a detailed description of the figures see the legend to Figure 3 in the main text.
(TIF)

Text S1 **Parameter settings of pre-processing algorithms for peak detection and quantification; reproducibility between the three average spectrum-dependent algorithms and ProteinChip Software 3.2.1 and Biomarker Wizard.**
(DOC)
