# Supplementary figures and images for: Reproducible Cancer Biomarker Discovery in SELDI-TOF MS Using Different Pre-Processing Algorithms
Source: PLoS One. 2011 Oct 14;6(10):e26294. doi: 10.1371/journal.pone.0026294 (PMC3194809; doi:10.1371/journal.pone.0026294)

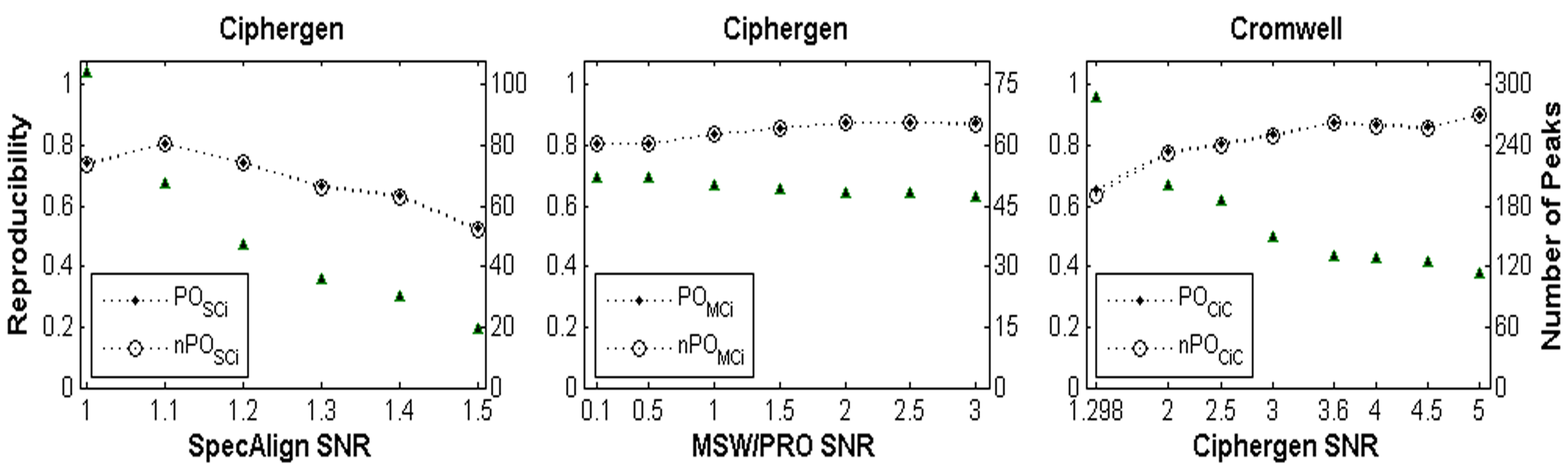

Supplement: Figure S1 — Reproducibility of peak detection between the average spectrum-dependent algorithms and Ciphergen for the breast cancer dataset. The reproducibility was evaluated between one algorithm (x-axis label) with various SNRs and another (title) with the default SNR. The default SNRs for SpecAlign, MSW/PRO, Cromwell and Ciphergen were 1.5, 3, 5 and 5, respectively. The filled triangles represent the number of peaks (right y-axis) detected by the algorithm shown by the x-axis label. All PO (nPO) scores were significantly higher than expected by chance (p<7.5E-12). (TIF) [file pone.0026294.s001.tif]

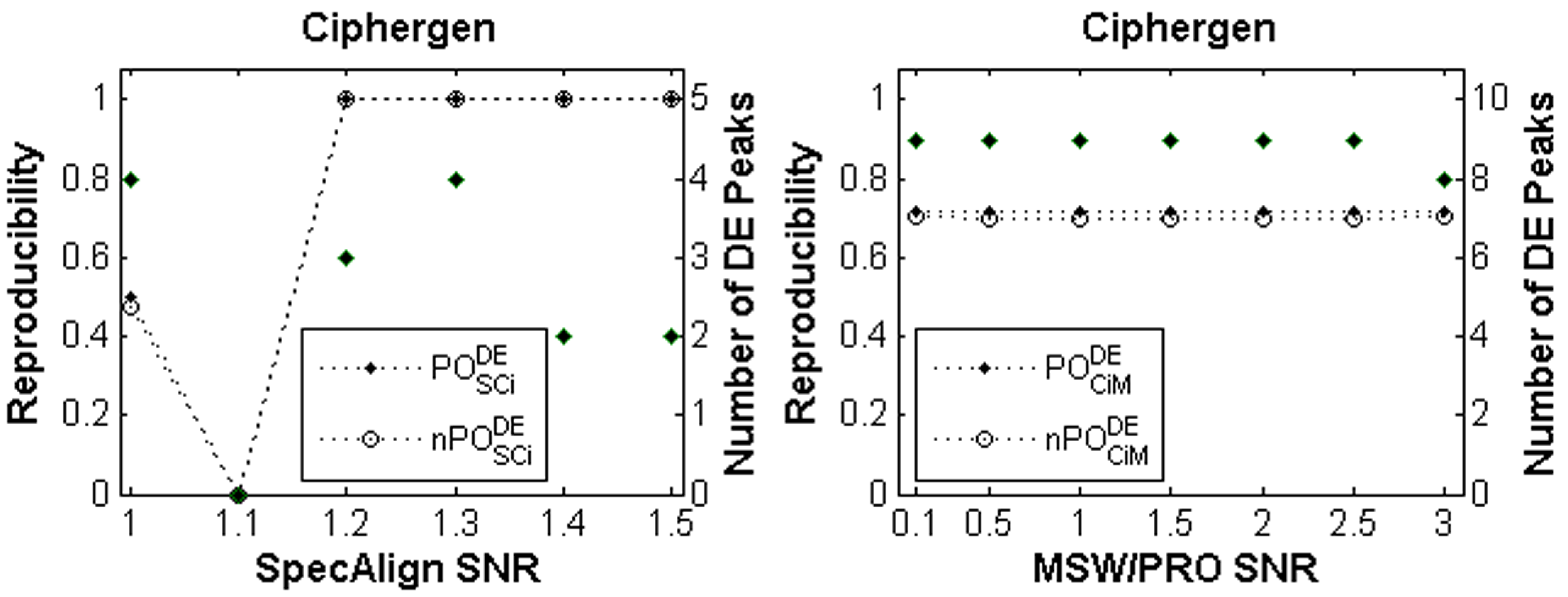

Supplement: Figure S2 — Reproducibility of DE peak detection between the average spectrum-dependent algorithms and Ciphergen for the breast cancer dataset. For Ciphergen with the default SNR, the stratified FDR control approach detected 7 DE peaks at the level of 10%. For SpecAlign and MSW/PRO, the simple FDR control approach was used to select DE peaks. All PODE (ncPODE) scores were significantly higher than expected by chance (p<9.0E-3). For a detailed description of the figures see the legend to Figure 3 in the main text. (TIF) [file pone.0026294.s002.tif]
