# Supplementary material for: Reproducible Cancer Biomarker Discovery in SELDI-TOF MS Using Different Pre-Processing Algorithms
Source: PLoS One. 2011 Oct 14;6(10):e26294. doi: 10.1371/journal.pone.0026294 (PMC3194809; doi:10.1371/journal.pone.0026294)
Supplement: Text S1 — Parameter settings of pre-processing algorithms for peak detection and quantification; reproducibility between the three average spectrum-dependent algorithms and ProteinChip Software 3.2.1 and Biomarker Wizard. (DOC) [file pone.0026294.s003.doc]

**Supporting Methods and Results**

**Supporting Methods**

**Parameter settings of pre-processing algorithms for peak detection and quantification**

**SpecAlign**

1. Processing\Smooth…


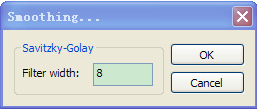


2. View\Baseline


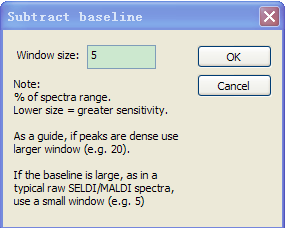


3. Processing\Subtract baseline

4. Processing\Rescale to positive

5. Processing\Normalize TIC


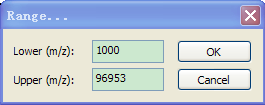


6. Spectrum\Generate average spectrum

7. Processing\Spectra alignment\FFT/Peak matching combined method


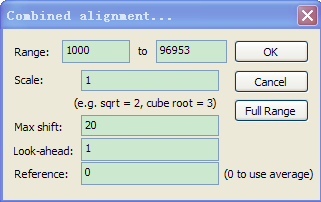


8. Spectrum\Pick peaks…


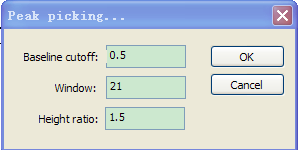


**MSW/PROcess**

1. PROcess for quantification

cutoff1 <- 1000

cutoff2 <- 10000

bldata <- rmBaseline1(mz,ms,method="approx",SpecNames=id)

blnormdata <- renorm(bldata,cutoff=cutoff1)

mznorm <- mz[which(mz>cutoff1)]

2. MassSpecWavelet for peak detection

SoN <- 3 # default signal-to-noise ratio

scales = c(1, seq(2, 30, 2), seq(32, 64, 4))

SNR.Th <- SoN

yi <- rowMeans(exp1) # Averaging the spectrum

yi_wCoefs <- cwt(yi, scales = scales, wavelet = "mexh")

yi_wCoefs <- cbind(as.vector(yi), yi_wCoefs)

colnames(yi_wCoefs) <- c(0, scales)

yi_localMax <- getLocalMaximumCWT(yi_wCoefs)

yi_ridgeList <- getRidge(yi_localMax)

yi_majorPeakInfo <- identifyMajorPeaks(yi, yi_ridgeList, yi_wCoefs, SNR.Th=SNR.Th)

yi_peakIndex <- yi_majorPeakInfo$peakIndex

peakmz <- mz[yi_majorPeakInfo$peakIndex]

peakmz <- peakmz[which(peakmz>=cutoff1 & peakmz<=cutoff2)]

intensityPeak <- matrix(NA,length(peakmz), ncol(exp1))

intensityPeak <- getPeaks2(blnormdata, peakmz, eps = 0.003)

profile <- cbind(peakmz,intensityPeak)

**Cromwell**

1. Compute the average spectrum

avg_spectrum = mean(raw_interp,2);

2. Denoise the average spectrum and find peaks

spectrumCutoffIndex = 1000;

waveletThreshold = 15;

[baseline_corrected_avg,smoothed_avg] = ...

waveletSmoothAndBaselineCorrect(avg_spectrum',...

waveletThreshold,...

spectrumCutoffIndex);

[peak_indices,left_peak_edge,right_peak_edge] = ...

trivialPeakFinder(baseline_corrected_avg);

3. Baseline correct individual spectra and extract peak heights

corrected_intensity = raw_interp; % space allocation only

% about 1 sec/spectrum

for(i1 = 1:n_spectra)

[baseline_corrected_spectrum,smoothed_spectrum] = ...

waveletSmoothAndBaselineCorrect(raw_interp(:,i1)',...

waveletThreshold,...

spectrumCutoffIndex);

corrected_intensity(:,i1) = baseline_corrected_spectrum';

time_track = clock;

[i1 time_track(4:6)]

end

normalization_factors = ...

mean(corrected_intensity(spectrumCutoffIndex:end,:));

corrected_intensity = corrected_intensity ./ ...

repmat(normalization_factors,length(corrected_intensity),1);

n_peaks = length(peak_indices);

peak_matrix = zeros(n_peaks,n_spectra);

for(i1 = 1:n_peaks)

peak_matrix(i1,:) = ...

max(corrected_intensity(...

left_peak_edge(i1):right_peak_edge(i1),:),[],1);

end

**ProteinChip Software 3.2.1 and Biomarker Wizard from Ciphergen Biosystems (denoted as Ciphergen)**

Smoothing is based on a moving average filter. Baseline is estimated with a piecewise convex-hull. Intensities of individual spectra are normalized with the average total ion current of all the spectra. “First Pass” and “Second Pass” for controlling SNR in Biomarker Wizard tool are used to detect peaks for individual spectra and then Ciphergen clusters peaks within a given mass window across spectra corresponding to the same biological molecule. The default settings of “First Pass” and “Second Pass” are 5 and 2, respectively. In this study, we mainly tuned “First Pass”; meanwhile, “Second Pass” was set as 1 when “First Pass” was less than 3, and 2 for otherwise [1].

**Supporting Results**

**Reproducibility** **between the three average spectrum-dependent algorithms and ProteinChip Software 3.2.1 and Biomarker Wizard**

**Reproducibility of Peak Detection**

For the breast cancer dataset, when using the default SNR for each algorithm, Ciphergen detected 113 peaks, more than 19 and 47 peaks identified by SpecAlign and MSW/PRO while less than 287 peaks detected by Cromwell. Among these 113 peaks, 90% were included in the 287 peaks detected by Cromwell. When decreasing the SNR (“First Pass”) to 1.298, Ciphergen detected the same number of peaks (287) as Cromwell but the PO_CiC_ (*n*PO_CiC_) score decreased to 0.65 (0.64) (Supplementary Figure 1). The peaks detected by SpecAlign and MSW/PRO were less consistent with those detected by Ciphergen with the PO_MCi_ (*n*PO_MCi_) as 0.53 (0.52) and the PO_MCi_ (*n*PO_MCi_) score as 0.83 (0.83). Even decreasing the SNRs to the lowest values, SpecAlign and MSW/PRO still detected fewer peaks than Ciphergen. The PO_SCi_ (*n*PO_SCi_) score increased to 0.74 (0.74) while the PO_MCi_ (*n*PO_MCi_) score decreased slightly to 0.81 (0.80) (Supplementary Figure 1).

The above results suggested when using the default SNR for each algorithm, SpecAlign and MSW/PRO tend to be less sensitive in peak detection than Ciphergen, and most of the peaks detected by these algorithms tend to be also detected by Cromwell. Cromwell can still capture almost all peaks detected by SpecAlign and MSW/PRO and most of the peaks detected by Ciphergen when decreasing the SNRs of the later three less sensitive algorithms.

**Reproducibility of DE Peak Detection**

For the breast cancer dataset, with 10% FDR control, no DE peak was selected for the Ciphergen-profile. Using the stratified FDR control approach, all 113 peaks in the Ciphergen-profile produced using the default SNR were grouped into 2 groups and then 7 DE peaks were detected with the FDR control level of 10%. These 7 DE peaks included most of DE peaks selected for the SpecAlign-profile and 5 of them were included in those selected for the MSW/PRO-profile (Supplementary Figure 2). Notably, all these 7 DE peaks were included in the 16 DE peaks selected by the stratified FDR control approach for the Cromwell-profile produced using the default SNR. In addition, these 7 DE peaks included the total 5 DE peaks reported in the original study for the breast cancer dataset [2].

**References**

1. Cruz-Marcelo A, Guerra R, Vannucci M, Li Y, Lau CC, et al. (2008) Comparison of algorithms for pre-processing of SELDI-TOF mass spectrometry data. Bioinformatics 24: 2129-2136.

2. Pusztai L, Gregory BW, Baggerly KA, Peng B, Koomen J, et al. (2004) Pharmacoproteomic analysis of prechemotherapy and postchemotherapy plasma samples from patients receiving neoadjuvant or adjuvant chemotherapy for breast carcinoma. Cancer 100: 1814-1822.
